# Editorial: Zika Virus Research

**DOI:** 10.3389/fneur.2018.00168

**Published:** 2018-03-23

**Authors:** Rubén Bueno-Marí, Juan-Carlos Saiz, Oscar D. Salomón, Luis C. Villamil-Jiménez, Jorg Heukelbach, Carlos H. Alencar, Paul K. Armstrong, Paulo H. Rosado-de-Castro, Pedro M. Pimentel-Coelho

**Affiliations:** ^1^Departamento de Investigación y Desarrollo (I + D), Laboratorios Lokímica, Valencia, Spain; ^2^Department of Biotechnology, Instituto Nacional de Investigación y Tecnología Agraria y Alimentaria, Madrid, Spain; ^3^Instituto Nacional de Medicina Tropical, Puerto Iguazú, Argentina; ^4^Grupo de Epidemiología y Salud Pública, Universidad de La Salle, Bogota, Colombia; ^5^Department of Community Health, School of Medicine, Federal University of Ceará, Fortaleza, Brazil; ^6^College of Public Health, Medical and Veterinary Sciences, Division of Tropical Health and Medicine, James Cook University, Townsville, QLD, Australia; ^7^Communicable Disease Control Directorate, Western Australia Department of Health, Perth, WA, Australia; ^8^Instituto de Ciências Biomédicas, Universidade Federal do Rio de Janeiro, Rio de Janeiro, Brazil; ^9^Instituto D’Or de Pesquisa e Ensino, Rio de Janeiro, Brazil; ^10^Instituto de Biofísica Carlos Chagas Filho, Universidade Federal do Rio de Janeiro, Rio de Janeiro, Brazil

**Keywords:** Zika virus, arbovirus, public health, mosquitoes, epidemiology, microcephaly, Guillain–Barré syndrome, flavivirus

**Editorial on the Research Topic**

**Zika Virus Research**

The considerable number of viral infectious disease threats that have emerged, since the beginning of the twenty-first century has shown the need to dispose global and coordinated responses to fight properly and efficiently against them. Severe acute respiratory syndrome (2003), avian influenza in humans (2005), A(H1N1) pandemic influenza (2009), Middle East respiratory syndrome coronavirus (MERS-CoV) (2012 onward), and Ebola virus disease (2014–2015) are some of the most important examples (1). The latest emerging and devastating threat was Zika virus, an arbovirus that provoked more than 580,000 autochthonous suspected disease cases in the Americas between 2015 and 2018 (2). Most notably, Zika caused social and medical alarm due to the evidence of a causal link between Zika virus and several congenital injuries, like microcephaly, as well as due to its association with neurological disorders, such as Guillain–Barré syndrome in adults (3). In the framework of this global response and multistrategic approach, the purpose of this research topic was to provide a platform for the publication of updated information and high-quality research papers about control strategies, encompassing virological, entomological, and epidemiological data, in order to reach the triad of protagonists of transmission cycles (virus, mosquitoes, and humans). We received 30 manuscripts, of which 23 were accepted for publication after rigorous peer review processes between March 22, 2016 and October 6, 2017.

Baraka and Kweka and Vythilingam et al. highlight the need to focus the Zika problem not only in the Americas but also to pay attention to what could happen in Sub-Saharan Africa (original region of the virus) and Southeast Asia (where the two most important vectors are well distributed). Recently, some of these concerns have become reality, especially in Southeast Asia where several countries have reported autochthonous cases in the past months (4).

As occurs with the rest of arthropod-borne diseases, detailed knowledge of the biology, behavior, and genetic plasticity of vectors are essential to predict potential outbreaks. Muñoz et al. provided an interesting research regarding the predictability of the conditions conducive to Zika epidemics based on a reproduction number model of the two most important disease vectors, namely *Aedes aegypti* and *Aedes albopictus*, that can be concurrent in disease risk areas. According to this study, “conditions for the occurrence of the Zika epidemic at the beginning of 2015 could have been successfully predicted at least 1 month in advance for several Zika hotspots, and in particular for Northeast Brazil: the heart of the epidemic.” Similarly, Nejati et al. used several climatic and topographic data to model and forecast which areas may be most prone to the establishment of *Ae. albopictus* in Iran. This interesting investigation is supposed to be the first study in the country to determine the regional probability for the establishment of this invasive mosquito of major concern for public health. Zika virus–vector interactions were explored by Anglero-Rodriguez et al. who found several differences in the tropism of Zika virus in two different *Ae. aegypti* strains and compared how Zika and dengue viruses affect the transcriptome of the vector. Moreover, Hunter also emphasizes the importance of not limiting Zika problems to *Ae. aegypti* presence, since too much information is available in relation to other susceptible mosquitoes, although it is clear that more infectivity studies are needed.

Prevention and control strategies have also been deeply discussed in the topic. One of the most applied examples has been given by Millet et al. that exposed the epidemiological and entomological surveillance program against imported Zika cases in the city of Barcelona (Spain). Rather et al. also reviewed the results of preventive campaigns conducted in different parts of the world, which were based on different approaches like, for instance, avoiding unnecessary travel to infected areas and mosquito bites. They argue that these and other proactive measures could “be employed to effectively combat the epidemic transmission of the Zika virus.”

Definitely, congenital abnormalities have triggered an increasing interest in Zika virus by medical, social, and economic reasons. Rather et al. provided an interesting and deep review about the scientific evidence supporting a causal relationship between ZIKV infection during pregnancy and congenital abnormalities. Coming closer to specific congenital and neurological problems, Yuan et al. commented a very interesting point of view regarding the teratogenic effects of Zika virus and the role of the placenta as natural barrier against the infection (5). Additionally, highlighted findings of Zika virus in cerebrospinal fluid samples from adults with neurological symptoms have been reported by Acevedo et al. in Ecuador.

Different virological aspects have also been well analyzed in the topic. Valadão et al. reviewed the mechanisms by which RNA viruses are recognized by host cells, triggering intracellular stress pathways and inflammatory responses, and how *Flaviviridae* members developed several strategies to counteract these cellular mechanisms. Furthermore, Giri et al. studied the prevalence of intrinsic disorder in Zika virus proteome (African strain MR 766). Their results revealed abundant intrinsically disordered protein regions (IDPRs) in Zika virus polyprotein, capsid protein, and several non-structural proteins, which could play a role in the pathogenesis of the disease. In an interesting study of the mutational preference direction of Zika virus, Khrustalev et al. contributed with promising and useful data that may have further implications for vaccine development and for the understanding of the evolution of new Zika virus strains.

Short discussions by way of articles commentaries could not be missed in the topic. Craig et al. and Saiz et al. crossed interesting points of view regarding the review article by Saiz et al.

Finally, some of the most relevant sources of knowledge of the Zika Virus research topic reside in several excellent reviews. Munjal et al. gives a great overview of different advances in the development of therapies against Zika virus, while Shukla et al. focus their review on rapid detection strategies of the virus in order to have quick response mechanisms to fight efficiently against the disease and to prevent reaching epidemic levels. Wider approach has been given by Shankar et al., encompassing additional aspects related to virus genome, clinical manifestations, diagnosis, and vaccine development. The four classical steps of epidemic management (transmission, detection, control, and prevention) are also well addressed by Sharma and Lal. Mini reviews about Zika virus emergence at American and worldwide level have been also interestingly written by Fajardo et al. and Rather et al., respectively. With all this information, Saiz et al. elaborated a very pertinent compilation of data and information to arrange all that has been learned at virological, entomological, clinical, and epidemiological level, since the start of Zika epidemic in late 2015.

We want to thank all the authors and reviewers for their valuable contributions to this research topic, and we hope that this collection of reviews, commentaries, and original articles will be helpful for clinicians, researchers, and students seeking for information about Zika virus.

## Author Contributions

Main text has been redacted by RB-M. All authors revised and approved the Editorial.

## Conflict of Interest Statement

The authors declare that the research was conducted in the absence of any commercial or financial relationships that could be construed as a potential conflict of interest.
